# Benefits of Premaquick^®^ Combined Detection of IL-6/Total IGFBP-1/Native IGFBP-1 to Predict Preterm Delivery

**DOI:** 10.3390/jcm12175707

**Published:** 2023-09-01

**Authors:** Mathilde Pambet, Fanny Sirodot, Bruno Pereira, Romain Cahierc, Amélie Delabaere, Aurélie Comptour, Marion Rouzaire, Vincent Sapin, Denis Gallot

**Affiliations:** 1 CIC 1405 CRECHE Unit, INSERM, Obstetrics and Gynaecology Department, CHU Clermont-Ferrand, 63000 Clermont-Ferrand, France; 2 Biostatistics Unit, Direction de la Recherche Clinique et de l’Innovation (DRCI), CHU Clermont-Ferrand, 63000 Clermont-Ferrand, France; 3 CNRS, SIGMA Clermont, Institut Pascal, Université Clermont Auvergne, 63000 Clermont-Ferrand, France; 4 Biochemistry & Molecular Genetic Department, CHU Clermont-Ferrand, 63000 Clermont-Ferrand, France; 5 “Translational Approach to Epithelial Injury and Repair” Team, Auvergne University, CNRS 6293, INSERM 1103, GReD, 63000 Clermont-Ferrand, France

**Keywords:** preterm labor, IGFBP-1, IL-6, fibronectin, vaginal biomarkers

## Abstract

We conducted a prospective double-blind study to compare two vaginal diagnostic methods in singleton pregnancies with threatened preterm labor (TPL) at the University Hospital of Clermont-Ferrand (France) from August 2018 to December 2020. Our main objective was to compare the diagnostic capacity at admission, in terms of positive predictive value (PPV) and negative predictive value (NPV), of Premaquick^®^ (combined detection of IL-6/total IGFBP-1/native IGFBP-1) and QuikCheck fFN™ (fetal fibronectin) for delivery within 7 days in cases of TPL. We included 193 patients. Premaquick^®^ had a sensitivity close to 89%, equivalent to QuikCheck fFN™, but a higher statistical specificity of 49.5% against 38.6% for QuikCheck fFN™. We found no superiority of Premaquick^®^ over QuickCheck fFN™ in terms of PPV (6.6% vs. 7.9%), with NPV being equivalent in predicting childbirth within 7 days in cases of TPL (98.6% vs. 98.9%). Nevertheless, the combination of positive native and total IGFBP-1 and the combination of all three positive markers were associated with a higher PPV. Our results, though non-significant, support this combined multiple-biomarker approach to improve testing in terms of predictive values.

## 1. Introduction

According to the latest national perinatal health survey published in 2021, 4.8% of women living in metropolitan France are hospitalized during their pregnancy for threatened preterm labor (TPL) [1]. This threat is the leading cause of hospitalization during pregnancy. TPL management [2] is based on hospitalization in a suitable care unit for gestational age, tocolysis, and corticosteroids for fetal lung maturation. Ambulatory care is acceptable in certain situations judged less critical. More than 50% of patients who present with symptoms and are admitted finally give birth at term. The clinical diagnosis of preterm labor (uterine contractions, cervical dilation up to 2 cm, 80% effacement) has up to a 50% false-positive rate, resulting in many patients being admitted and receiving unnecessary prophylactic treatments [3]. They can potentially cause adverse maternal and neonatal effects; for example, tocolytics can cause adverse drug reactions in mothers, and unnecessary antenatal corticosteroids are associated with adverse neurodevelopmental outcomes in children [4]. Moreover, it results in a substantial economic burden to health services and negative financial and emotional impacts on women and families [5]. 

TPL is a real public health issue. Obstetricians need reliable tools to assess the real risk of a premature birth. Today, this assessment is based on the ultrasound measurement of cervix length, in particular, to predict childbirth within 7 days [6]. Various biological markers present in vaginal secretions can serve as predictors of preterm birth, such as fibronectin and cytokines interleukin-6 (IL-6), -8 (IL-8), -10 (IL-10), or TNF-α (tumor necrosis factor) [7,8,9,10,11]. Insulin-like growth factor-binding protein-1 (IGFBP-1), whose presence in vaginal secretions is well known in the context of premature rupture of membranes (PROM), can also be an important predictor for premature delivery [12,13].

Nevertheless, most of the biological diagnostic tests used to detect TPL are qualitative tests that detect fetal fibronectin (fFN) from cervico-vaginal secretions via immunochromatographic assay. Of these, the QuikCheck fFN™ (fetal fibronectin) test (Hologic) has a negative predictive value (NPV) of approximately 99% for no childbirth within 7 days and about 96–97% for no childbirth within 14 days [14]. Owing to its low positive predictive value (PPV), the usefulness of this test, added to ultrasound measurement of the cervix, is in doubt. The combined detection of IL-6 and IGFBP-1, total (native plus fragmented) and native (non-fragmented form), is proposed in a new predictive test for preterm birth, Premaquick^®^ (Biosynex, Strasbourg, France). IGFBP-1 is a marker of cervical ripening. Its presence in vaginal secretions in the absence of ruptured membranes indicates a significant lysis of the decidual cells of the cervix and a diffusion of amniotic fluid during contractions. The presence of fragmented forms of IGFBP-1 reflects a significant local proteolytic activity and fetal stress caused by contractions. The third marker, IL-6, signals inflammation or infection of the amniotic cavity and the cervicovaginal area. Combining the biomarkers of myometrium activity, ripening of the cervix and inflammation/infection, Premaquick^®^ encompasses the principal pathogenic mechanisms for preterm labor. One study found that this test had an NPV comparable to Quikcheck fFN™ [15]. It was 98% for predicting ongoing pregnancy within 7 and 14 days when all three markers were negative. Its PPV was 84% and 94% to predict delivery within 7 or 14 days, respectively, when at least two markers were positive. It rose to 95.8% to predict delivery within 7 or 14 days when all three markers were positive. More recently, another Nigerian team found that QuikCheck fFN™ had a higher specificity (98.5% vs. 97.8%, *p* > 0.99) but Premaquick^®^ had a higher PPV (92.7% vs. 90.9%; *p* > 0.99) [16]. Another team found that Premaquick^®^ had 95.1% sensitivity, 97.5% specificity, 97.5% positive predictive value, 95.2% negative predictive value, and 96.3% overall accuracy in predicting premature birth [17].

Our main hypothesis was that the combined detection of IL-6, total IGFBP-1, and native IGFBP-1 on admission would improve the prediction of premature birth (in terms of PPV and NPV) in cases of TPL. Our main objective was to compare the ability of Premaquick^®^ and QuikCheck fFN™ to predict delivery within 7 days from admission in the event of TPL in a population with an ultrasound cervical length < 30 mm. Our secondary objectives were to assess the ability of these tests to predict an unfavorable outcome within 14 days (delivery within 14 days, PROM, re-hospitalization, resumption of tocolytic treatment) and to evaluate the diagnostic capacity of Premaquick^®^ in terms of PPV and NPV according to the number of positive or negative markers (IL-6/total IGFBP-1/native IGFBP-1).

## 2. Materials and Methods

### 2.1. Design

This was an exploratory double-blind study comparing two diagnostic methods in the context of TPL: QuikCheck fFN™ vs. Premaquick^®^. Patients were included consecutively at the University Hospital of Clermont-Ferrand, France.

### 2.2. Population

The inclusion criteria were age over 18 years and admission for TPL with intact membranes between 24 + 0 GW and 34 + 6 GW. TPL was defined as symptomatic uterine contractions documented on an external cardiotocographic recording associated with a shortened cervix (ultrasound cervical length < 30 mm). Patients were covered by French social security and gave their informed consent.

Non-inclusion criteria were the presence of cervical dilation ≥ 4 cm, multiple pregnancy, ROM, uterine malformation, polyhydramnios, fetal malformation, placenta previa, profuse bleeding, earlier participation in the study for a previous episode of TPL during the same pregnancy, or if the patient was under guardianship or curatorship. 

### 2.3. Study Procedures and Data Collected

Enrolled patients underwent double simultaneous swab vaginal secretion sampling at the level of the posterior vaginal fornix under speculum. They were assessed for temperature, hemogram, CRP level, and urine and vaginal bacteriological culture. The usual management for TPL was unchanged (±admission, ±corticosteroids for lung maturation, ±tocolysis, and ±etiological treatment). Antenatal corticosteroids for fetal maturation were administered according to the national guidelines: betamethasone given as two 12 mg intramuscular injections 24 h apart. Data were collected and managed using the REDCap (Research Electronic Data Capture) tool hosted at the University Hospital of Clermont-Ferrand [18]. REDCap is a secure, web-based software platform designed to support data capture for research studies.

### 2.4. Description of the Tests 

The immunochromatographic tests for (i) fetal fibronectin detection (QuikCheck fFN™) and (ii) IL-6, total IGFBP-1, and native IGFBP-1 (Premaquick^®^) were carried out by technicians at the central laboratory, following the supplier’s instructions (see Supplementary Files S1 and S2). 

For QuikCheck fFN™, a negative result indicating the absence of fetal fibronectin appeared as one line. A positive result indicating the presence of fetal fibronectin appeared as two lines (Supplementary File S1).

For Premaquick©, the results were interpreted as negative, positive, or invalid, depending on the presence of colored bands in the procedure control area (C) and test areas (T) for the three markers. The presence of three control bands (C) in the result reading windows was a condition for the test to be valid. The presence or absence of the test band T (even when of low intensity) for each parameter was scored as follows: 3 for a test band T for the IGFBP-1 N marker, 2 for a test band T for the IGFBP-1 marker, 1 for a test band T for the IL-6 marker, and 0 for no test band T. The scores were summed to give the total score (see Supplementary File S2). A total score equal to or less than 2 meant a high probability of birth within 7–14 days.

The test results were not disclosed to the clinician or the patient before delivery and so did not affect management.

### 2.5. Statistical Analysis

The objective of this study was to compare positive and negative predictive values of Premaquick© (combined detection of IL-6/total IGFBP-1/native IGFBP-1) and Quikcheck fFN™ (detection of fibronectin) at admission to predict delivery within 7 days in cases of threatened preterm birth. More precisely, a non-inferiority assumption was proposed for NPV, whereas a difference for PPV was expected. It was proposed to include 200 patients in order to guarantee a satisfactory statistical power to highlight (i) a non-inferiority limit of 4% concerning the negative predictive value for 99% expected NPV for the detection of fibronectin (Quikcheck fFN™) with at least 95% statistical power and one-sided type I error at 5%, i.e., 170 patients for an intra-individual correlation at 0.5, and (ii) an absolute difference of at least 20% for the following PPV values—50% for fibronectin detection (Quikcheck fFN™) and 70% for the Premaquick© test) for a power greater than 80%, i.e., 56 patients for an intra-individual correlation at 0.5. The continuous variables were expressed as mean and standard-deviation or as median and interquartile range. The normal distribution assumption was checked using the Shapiro–Wilk test. To compare the diagnostic capacity on admission of Premaquick^®^ and QuikCheck fFN™ for delivery within 7 days in cases of TPL, the primary analysis was based on (i) the McNemar test for paired proportions for PPV and (ii) an analysis of the 95% confidence interval for the non-inferiority study of NPV. The same statistical analyses were conducted to evaluate the diagnostic ability to predict an unfavorable 14-day outcome in terms of delivery, PROM, re-hospitalization, and repeat tocolytic therapy. Comparisons of continuous variables (such as age, gestational age at inclusion, BMI) between Premaquick^®^ and QuikCheck fFN™ results were made using Student’s *t*-test or the Mann–Whitney test if the criteria for applying the *t*-test were not met. The equality of variances was analyzed with the Fisher–Snedecor test. For categorical variables (such as marital status, gestity, parity, smoking), chi-squared or Fisher’s exact tests were used. Statistical analyses were performed with Stata software (version 15; StataCorp, College Station, TX, USA) for a two-sided type I error of 5%.

## 3. Results

We included 193 singleton pregnancies between 21 August 2018 and 21 December 2020. All included patients underwent a double simultaneous swab with Premaquick^®^ and QuikCheck fFN™. 

Population characteristics and obstetric history are listed in Table 1. None of these variables had any statistically significant impact on the positivity of Premaquick^®^ or QuikCheck fFN™ (*p* > 0.05).

Hemodynamic constants and temperature were normal in all the patients. The number of uterine contractions per 10 min was greater when Premaquick^®^ and QuikCheck fFN™ were positive. This difference was statistically significant for the two tests, with, respectively, *p* = 0.04 and *p* = 0.02 (Table 2).

In the 160 patients admitted, the mean hospital stay was 3.98 ± 2.5 days. In 79% of cases, weekly or bi-weekly monitoring was set up by a home midwife; 81.1% of included patients underwent a fetal lung maturation cure via an injection of betamethasone. There was no significant difference in the proportion of women who received betamethasone between patients with a positive or negative test result (*p* = 0.17 for Premaquick^®^ or *p* = 0.55 for QuikCheck fFN™).

Tocolysis was required in 72.5% of cases. It was administered orally in 71.4% of cases (nifedipine) and intravenously in 28.6% of cases (atosiban) (Table 2). The mean duration of tocolytic treatment was 2.2 ± 1.37 days. 

**Figure 1:**
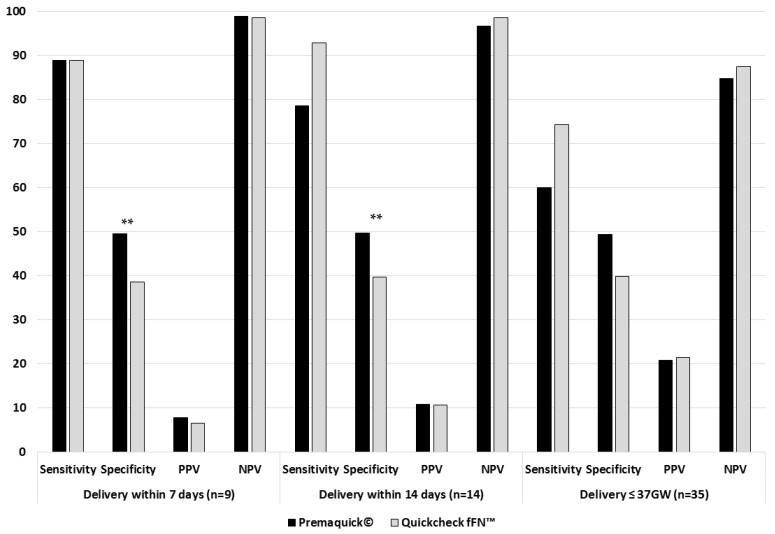
Diagnostic capacity of Premaquick^®^ and QuikCheck fFN™. ** mean *p*-value < 0.01.

In our study, nine (4.7%) patients gave birth within 7 days. Premaquick^®^ was positive for eight of these test patients, as was QuikCheck fFN™. The PPV of Premaquick^®^ to predict a premature delivery within 7 days in cases of TPL was 7.9% with an NPV of 98.9%, a sensitivity of 88.9%, and a specificity of 49.5%. For QuikCheck fFN™, 7-day PPV was 6.6% with an NPV of 98.6%, a sensitivity of 88.9%, and a specificity of 38.6% (Figure 1). Premaquick^®^ was non-inferior in terms of NPV compared with QuikCheck fFN™ (difference between NPV values: 0.3% [−2.6%; 3.2%], *p* = 0.86), but we found no difference in PPV (*p* = 0.71).

For our secondary objectives, 14 (7.3%) patients gave birth within 14 days; 13 had a positive fetal fibronectin test, and 11 had a positive Premaquick^®^ test. The PPV of Premaquick^®^ to predict a premature delivery within 14 days in the event of TPL was 10.9% with an NPV of 96.7%, a sensitivity of 78.6%, and a specificity of 49.7%. For QuikCheck fFN™, PPV was 10.7% with an NPV of 98.6%, a sensitivity of 92.9%, and a specificity of 39.7% (Figure 1).

Of the patients, 35 (18.1%) gave birth before 37 weeks; 26 had a positive fetal fibronectin test, and 21 had a positive Premaquick^®^ test. The PPV of Premaquick^®^ to predict a premature delivery before 37 weeks in cases of TPL was 20.8% with an NPV of 84.8%, a sensitivity of 60%, and a specificity of 49.4%. For Quikcheck fFN™, PPV was 21.5% with an NPV of 87.5%, a sensitivity of 74.3%, and a specificity of 39.9% (Figure 1). 

No difference was found for other secondary outcomes: six patients presented a PROM within 14 days of their inclusion. These two tests were not statistically significant in predicting the premature rupture of membranes within 14 days (*p* = 0.12 for Premaquick^®^ and *p* = 0.84 for QuikCheck fFN™). In our study, the mean time to re-hospitalization was 19 days; 17 patients required re-hospitalization for TPL within 14 days of inclusion; 14 had a positive fetal fibronectin test (*p* = 0.08) and eight had a positive Premaquick^®^ test (*p* = 0.65). These two tests were not statistically significant in predicting re-hospitalization within 14 days. Statistically significant results were not found for predicting the repetition of tocolytic treatment within 14 days. Only 21 patients received a new tocolysis within 14 days; 16 patients had a positive fetal fibronectin test (*p* = 0.18), and 10 had a positive Premaquick^®^ test (*p* = 0.65). 

We studied the diagnostic capacity of Premaquick^®^ based on the number of positive markers for delivery within 7 days, 14 days, and before 37 GW. Among the eight possible combinations (see Supplementary File), six were represented in our study, excluding isolated native IGFBP-1 and native IGFBP-1 plus IL-6. All SE, SP, PPV, and NPV results are presented in Table 3. Only three patients were positive for both total and native IGFBP-1 and negative for IL-6. 

When all three markers were positive, which was the case for 13 patients, there were three deliveries in 7 days, four in 14 days, and four before 37 GW. The PPV was 23.1% for a delivery in 7 days (vs. 7.9% for positive Premaquick^®^ according to the manufacturer’s score, *p* = 0.11), 30.8% for a delivery in 14 days (vs. 10.9%, *p* = 0.07), and 30.8% before 37 weeks (vs. 20.8%, *p* = 0.48). NPV was 96.7% for predicting a delivery within 7 days (vs. 98.9% for positive Premaquick^®^ K, *p* = 0.43), 94.4% for a delivery in 14 days (vs. 96.7%, *p* = 0.55), and 82.8% for a delivery before 37 weeks (vs. 84.8%, *p* = 0.67).

## 4. Discussion

In our study, 158 (81.9%) patients treated for TPL gave birth after 37 weeks. This is in line with data classically published in the literature [1,19,20]. According to the latest national perinatal health survey published in 2021, more than 50% of patients hospitalized for TPL ultimately give birth at term [1]. The data from our study did not show any difference in terms of PPV for Premaquick^®^ and Quikcheck fFN™ to predict a premature delivery within 7 days in cases of TPL (7.9% vs. 6.6%). In terms of NPV, there was also a non-inferiority of Premaquick^®^ compared with Quikcheck fFN™ (98.9% vs. 98.6%). In the study of Eleje et al. [15], 97 patients with TPL were analyzed (singleton pregnancy only): 6 patients gave birth within 7 days and 8 within 14 days. The authors found a PPV and NPV of 70.5% and 100%, respectively, within 7 days. Within 14 days, the PPV was 87.5% and the NPV was 95%. They also studied the diagnostic ability of the test for a triple positive or negative. When all three markers were positive, they found 95.8% PPV and 98% NPV within 7 and 14 days, against our 23.1% and 30.8% PPV and 96.7% and 94.4% NPV. In a more recent Nigerian study, [16] 183 women were enrolled and 175 completed the study. The sensitivity, specificity, PPV, NPV, and accuracy of the Premaquick^®^ versus fFN tests were, respectively, 96.3% vs. 51.9%, 97.6% vs. 98.4%, 89.7% vs. 87.5%, 99.2% vs. 90.3%, and 97.3% vs. 90.0% for preterm delivery within 14 days; fFN had higher specificity (98.5% vs. 97.8%; *p* > 0.99), but Premaquick^®^ had higher PPV (92.7% vs. 90.9%, *p* > 0.99). Our results conflict with the PPVs and NPVs found in these two studies, which nevertheless present a prematurity rate close to ours. We hypothesize that this difference is due to the evaluation methods used for the tests. In our study, the analysis of the results was carried out in a centralized and standardized mode in a biochemistry laboratory, blinded to the clinician and the patient, to avoid a potential bias of evaluation that can occur when the tests are performed at the patient’s bedside.

Another team compared 122 pregnant women admitted for TPL before 37 weeks with 122 controls to evaluate the accuracy of the Premaquick^®^ test in detecting TPL [17]. They found 95.1% sensitivity, 97.5% specificity, 97.5% PPV, 95.2% NPV, and 96.3% accuracy in detection of TPL. Abu-Faza et al., who included 110 women with TPL and 110 controls in the study group, found lower sensitivity (39.8%) and NPV (62.2%) [21]. These results cannot be compared with ours because of the design differences between the studies.

We examined the diagnostic capacity of each of the three biomarkers present in Premaquick^®^. Each of the markers had strong NPV but low PPV. Native IGFBP-1 was never positive alone. The literature reports many similar results for the diagnostic capacity of biomarkers, with strong NPVs but highly variable and often weak PPVs. In 2002, Lembet et al. studied the effectiveness in 36 patients of a rapid phosphorylated-IGFBP-1 detection test in vaginal secretions, the ActimPartus test, to predict premature delivery in cases of TPL. The PPV and NPV of this test were, respectively, 83.3% and 94.1% to predict delivery within 7 days [22]. Ting et al., in 2007, in a study similar to ours, compared the effectiveness of the rapid detection of IGFBP-1 in its phosphorylated form with a test for the detection of fetal fibronectin. Both tests had strong NPVs for predicting premature childbirth within 2, 7 and 14 days. The IGFBP-1 test had PPVs under 2, 7 and 14 days at 18%, 39% and 46%, respectively [23]. These values came close to our results in the triple-positive Premaquick^®^ test case (23.1%, 30.8%, and 30.8%). For the detection of IGFBP-1, Cooper et al., in 2011, found a PPV of 24% to predict premature delivery < 37 GW. Compared with fetal fibronectin, like in our analysis (PPV 21.5%), no difference was observed [24].

We demonstrated a statistically significant difference concerning cervical length measured using ultrasound for a positive or negative Premaquick© test (*p* = 0.05) but not for the Quikcheck fFN™ test (*p* = 0.47). Since 2010, French official guidelines have recommended cervix measurement via endovaginal ultrasound to identify patients requiring specific care, with either TPL or identified risk factors for preterm delivery [25]. Kumari et al. showed that the detection of IGFBP-1 in vaginal secretions combined with ultrasound measurement of cervical length was more effective at predicting premature delivery in cases of TPL than the detection of IGFBP-1 alone (VPN at 96% approximately versus 93%) [26]. In contrast, the combination of these tests did not influence the PPV, which remained low: 29% within 2 days, 33.5% within 7 days, and 34% within 14 days [27]. In our analysis, we found an equivalent PPV to predict childbirth < 37 GW (approximately 23.8%) when total IGFBP-1 was positive.

The occurrence of TPL is multifactorial (socio-economic environment, maternal age, addictive behavior, multiple pregnancy, etc.), but associated infections and inflammation are often explored as they are theoretically accessible to prevention or therapy. We set out to study the usefulness of inflammation biomarkers including IL-6 to predict the risk of premature delivery. Both infectious and sterile inflammation have been clearly established as important actors linked to premature childbirth. IL-6 is rapidly and abundantly upregulated upon an infectious stressor and initiates an immune response mediated by neutrophil, macrophages and lymphocytes cells during preterm birth [28]. A study published in 1994 showed that the presence of IL-6 in vaginal secretions between 24 weeks and 36 weeks, in asymptomatic patients, was an independent predictor of preterm delivery (OR: 4.8 with 95% CI: 1.7–14.3). On the other hand, elevated IL-6 levels were not correlated with maternal infectious morbidity [27]. In 2003, Lange et al. analyzed the presence of IL-6 at elevated levels in vaginal secretions in 31 patients with TPL. To predict a premature delivery within 7 days, IL-6 (>20 pg/mL) had a sensitivity of 100% and specificity of 67%. IL-6 was therefore a promising biomarker for screening patients at high risk of premature delivery in cases of TPL [8]. In our analysis, we found a VPN of 94.7% to predict a delivery within 7 days and 91.8% for a delivery at 14 days. The highest PPV of IL-6 was 13.6% for predicting childbirth at <37 GW.

Many studies have looked at combinations of several biomarkers to improve the diagnostic capacity of these tests [29,30]. The strongest PPVs, under 7 days, 14 days and before 37 GW, were obtained for a triple-positive Premaquick©. It seemed logical to associate the measurement of cervical length with the detection of these biomarkers with the aim of identifying the patients most at risk of giving birth prematurely. In 2001, Kurkinen-Raty et al. published a prospective study to assess the advantage of combining the detection of IL-6, IL-8, and phosphorylated IGFBP-1 with the measurement of cervical length via ultrasound in 77 patients treated for TPL. The presence of elevated levels of IGFBP-1 and IL-8 and a cervical length < 29.3 mm increased the risk of premature delivery but not significantly [29]. The combination of markers IL-6 and IL-8 and a cervical ultrasound via the endovaginal route (length + funneling) had a specificity of 97% but a sensitivity of 30%. This was the best combination to predict a premature delivery with an OR of 4.3 (95% CI 1.0–19). High concentrations of IGFBP-1 in combination with IL-6, IL-8, and cervical ultrasound increased the risk of premature delivery (OR 4.3 with a 95% CI 1.0–19), with a specificity of 98%, but this combination reduced the sensitivity to 10%.

In 2011, Menon et al. published a meta-analysis on the biomarkers of spontaneous prematurity analyzed over the past 40 years. About 120 biomarkers were described among the 217 studies analyzed. Two-thirds of these studies were carried out in North America and in Europe. There was marked heterogeneity in the design of these studies. No biomarker emerged as a predictor of preterm delivery [30]. Likewise, in our analysis, none of the three biomarkers was found to outperform either of the others. 

We found no difference in terms of PPV for the two tests studied. Premaquick^®^ was more statistically specific for predicting premature delivery within 7 days than Quikcheck fFN™ (49.5% vs. 38.6%). The NPVs of Premaquick^®^ and Quikcheck fFN™ were strong and equivalent in predicting delivery within 7 and 14 days (98.9% vs. 98.6%). In the event of a negative Premaquick^®^, it would thus seem reasonable to consider ambulatory care and limit excessive treatment (tocolysis, corticosteroids). As the two tests performed similarly, cost may be a factor in the choice. 

Our study has several strengths. It was a prospective study, conducted in a double-blind fashion for both clinicians and patients, ensuring that clinical decisions at admission, such as orientation (hospitalization or ambulatory) and the use of antenatal betamethasone or tocolysis, were not impacted by the test results. All patients benefited from the same management according to the protocol of the obstetrics department of the University Hospital. No patients were lost to follow-up and there were very few missing data. A limitation of our study is that it was a single-center study. Another weak point of our analysis was the sample size (*n* = 193), with only nine patients who gave birth within 7 days.

## 5. Conclusions

We found no superiority of Premaquick© over Quikcheck fFN™ in terms of positive predictive value, with negative predictive value equivalent for predicting childbirth within 7 days in cases of TPL, and likewise for our secondary objectives within 14 days: childbirth, premature rupture of membranes, re-hospitalization, and repeat tocolytic treatment. Nevertheless, the combination of positive native and total IGFBP-1 and the combination of all three positive markers were associated with the highest PPV. Though non-significant, our results support multiple-marker approaches to improve the predictive capacity of biological diagnostic tests.

## Figures and Tables

**Table 1:** Characteristics of the population.

	All Patients	Premaquick^®^ +	Premaquick^®^ −	*p*	Quikcheck fFN™ +	Quikcheck fFN™ −	*p*
*n*	193	101	92		121	72	
Age at inclusion (years)	28.3 ± 5.3	28.2 ± 5.5	28.4 ± 5.2	0.82	28.2 ± 5.6	28.5 ± 4.8	0.68
Gestational age at inclusion (GW)	30.8 ± 2.7	30.6 ± 2.8	30.9 ± 2.5	0.53	30.6 ± 2.9	31.1 ± 2.4	0.21
BMI ^1^ (kg/m^2^) before pregnancy	23.3 ± 4.8	23.1 ± 4.6	23.44 ± 5.0	0.65	23.4 ± 4.8	23.05 ± 4.7	0.60
Marital status							
Single	14.6 (28)	11.0 (11)	18.5 (17)	0.14	14.2 (17)	15.3 (11)	0.83
In a relationship	85.4 (164)	89.0 (89)	81.5 (75)		85.8 (103)	84.7 (61)	
Gestation							
1	39.9 (77)	44.6 (45)	34.8 (32)	0.26	39.7 (48)	40.3 (29)	0.49
2	26.4 (51)	26.7 (27)	26.1 (24)		24.0 (29)	30.6 (22)	
≥3	33.7 (65)	28.7 (29)	39.1 (36)		36.3 (44)	29.2 (21)	
Parity							
0	50.8 (98)	54.5 (55)	46.7 (43)	0.24	47.9 (58)	55.6 (40)	0.50
1	15.0 (29)	16.8 (17)	13.0 (12)		14.9 (18)	15.3 (11)	
≥2	34.2 (66)	28.7 (29)	40.2 (37)		37.2 (45)	29.2 (21)	
Active smoking							
Before pregnancy	32.1 (62)	32.7 (33)	31.5 (29)	0.86	36.4 (44)	25.0 (18)	0.10
During pregnancy	21.2 (41)	21.8 (22)	20.6 (19)	0.85	24.0 (29)	16.7 (12)	0.23
Gestational diabetes	10.9 (21)	8.0 (8)	14.1 (13)	0.17	11.7 (14)	9.7 (7)	0.68
Sexual intercourse < 24 h at inclusion	12.1 (21)	14.6 (13)	9.4 (8)	0.29	12.5 (14)	11.3 (7)	0.81
History (previous pregnacies)							
TPL	18.7 (36)	20.8 (21)	16.3 (15)	0.42	19.8 (24)	16.7 (12)	0.58
Preterm birth	15.5 (30)	13.9 (14)	17.4 (16)	0.50	16.3 (20)	13.9 (10)	0.62
ROM ^2^	4.2 (8)	5.0 (5)	3.3 (3)	0.56	4.1 (5)	4.2 (3)	0.99
Cervical surgery	2.1 (4)	2.0% (2)	2.2% (2)	0.92	2.5 (3)	1.4 (1)	0.607

^1^ BMI—body mass index (kg/m^2^); ^2^ ROM**—**rupture of membrane.

**Table 2:** Patient assessment and management.

	All Patients	Premaquick^®^ +	Premaquick^®^ −	*p*	Quikcheck fFN™ +	Quikcheck fFN™ −	*p*
*n*	193	101	92		121	72	
Number of UC/10 min	1.70± 1.50	1.88 ± 1.60	1.44 ± 1.42	0.04	1.86 ± 1.58	1.36 ± 1.40	0.03
Cervical length (mm)	18.9 ± 5.9	18.1 ± 6.2	19.8 ± 5.5	0.05	18.7 ± 6.2	19.3 ± 5.5	0.47
Orientation							
Hospitalization *	82.9 (160)	85.2 (86)	80.4 (74)	0.38	81.0 (98)	86.1 (62)	0.36
Use of antenatal betamethasone	81.2 (155)	84.9 (84)	77.2 (71)	0.17	79.8 (95)	83.3 (60)	0.55
Tocolysis **	28.6 (40)	34.2 (26)	21.9 (14)	0.11	33.3 (30)	20.0 (10)	0.09

* Versus ambulatory; ** versus per os (nifedipine). UC—uterine contraction.

**Table 3:** Diagnostic capacity of Premaquick^®^ according to the number of markers testing positive. Sensitivity/specificity/positive predictive value/negative predictive value.

IL-6	IGFBP-1 Total	IGFBP-1 Native	*n* (+)	Delivery in 7 Days (*n* = 9)	Delivery in 14 Days (*n* = 14)	Delivery before 37 GW (*n* = 34)
−	−	−	67	11.1/64.1/1.5/93.7	21.4/64.2/4.5/91.3	28.6/63.9/14.9/80.2
−	+	−	45	33.3/77.2/6.7/95.9	21.4/76.5/6.7/92.9	17.1/75.3/13.3/80.4
+	−	−	22	0.0/88.0/0.0/94.7	0.0/87.7/0.0/91.8	8.6/88.0/13.6/81.3
+	+	−	42	11.1/77.7/2.4/94.7	21.4/78.2/7.1/92.7	28.6/79.7/23.8/83.4
−	+	+	3	11.1/98.9/33.3/95.8	7.1/98.9/33.3/93.2	2.9/98.7/33.3/82.2
+	+	+	13	33.3/94.6/23.1/96.7	28.6/95.0/30.8/94.4	11.4/94.3/30.8/82.8
Positive Premaquick^®^ *				**88.9/49.5/7.9/98.9**	**78.6/49.7/10.9/96.7**	**60.0/49.4/20.8/84.8**
Positive QuikCheck™ *				88.9/38.6/6.6/98.6	92.9/39.7/10.7/98.6	74.3/39.9/21.5/87.5

* Tests were considered positive according to the manufacturer’s score (see Supplementary File S1 for Quikcheck™ and Supplementary File S2 for Premaquick^®^).

## Data Availability

The data presented in this study are available on request from the corresponding author.

## Supplementary Materials

The following supporting information can be downloaded at: https://www.mdpi.com/article/10.3390/jcm12175707/s1.
