# Supplementary material for: Benefits of Premaquick® Combined Detection of IL-6/Total IGFBP-1/Native IGFBP-1 to Predict Preterm Delivery
Source: J Clin Med. 2023 Sep 1;12(17):5707. doi: 10.3390/jcm12175707 (PMC10488604; doi:10.3390/jcm12175707)
Supplement: Supplementary file 1 [file jcm-12-05707-s001.zip › Supplementary File S1.pdf]

HOLOGIC®

QuikCheck fFN™ Test Kit

REF 01270 REF PRD-01864

For *In Vitro* Diagnostic Use Only
Store at 2° to 25°C. Do not freeze.

CE

The Hologic QuikCheck™ fFN is a qualitative test for the detection of fetal fibronectin in cervicovaginal secretions.
For Professional Use Only

INTENDED USE

The Hologic QuikCheck fFN test is intended to be used for the qualitative detection of fetal fibronectin in cervicovaginal secretions. The presence of fetal fibronectin in cervicovaginal secretions between 22 weeks, 0 days and 34 weeks, 6 days of gestation is associated with elevated risk of preterm delivery.

SUMMARY AND EXPLANATION OF THE TEST

Preterm delivery, defined by the American College of Obstetricians and Gynecologists as delivery prior to the 37th week of gestation, is responsible for the majority of non-chromosomal perinatal morbidity and mortality (1–4). Symptoms of threatened preterm delivery include uterine contractions, change of vaginal discharge, vaginal bleeding, backache, abdominal discomfort, pelvic pressure, and cramping. Diagnostic modalities for identification of threatened preterm delivery include uterine activity monitoring and performance of a digital cervical examination, which allows estimation of cervical dimensions. These methods have been shown to be limited, as minimal cervical dilatation (< 3 centimeters) and uterine activity occur normally and are not necessarily diagnostic of imminent preterm delivery (5,11,13). While several serum biochemical markers have been evaluated, none have been widely accepted for practical clinical use (6,7,20).

Fetal fibronectin (fFN), an isoform of fibronectin, is a complex adhesive glycoprotein with a molecular weight of approximately 500,000 daltons (8,9). Matsuura and co-workers have described a monoclonal antibody called FDC-6, which specifically recognizes III-CS, the region defining the fetal isoform of fibronectin (8,9). Immunohistochemical studies of placentae have shown that fFN is confined to the extracellular matrix of the region defining the junction of the maternal and fetal units within the uterus (5,10).

Fetal fibronectin can be detected in cervicovaginal secretions of women throughout pregnancy by use of a monoclonal antibody-based immunoassay. Fetal fibronectin is elevated in cervicovaginal secretions during early pregnancy but is diminished from 22 to 35 weeks in normal pregnancies. The significance of its presence in the vagina during the early weeks of pregnancy is not understood. However, it may simply reflect the normal growth of the extravillous trophoblast population and the placenta. Detection of fFN in cervicovaginal secretions between 22 weeks, 0 days and 34 weeks, 6 days gestation is reported to be associated with preterm delivery in symptomatic (5,11–15) and between 22 weeks, 0 days and 30 weeks, 6 days in asymptomatic pregnant women (16-19).

PRINCIPLE OF THE TEST

The QuikCheck fFN is a solid-phase immunogold assay. Specimens obtained from the posterior fornix are placed into an extraction buffer. A test strip with immobilized mouse monoclonal anti-fetal fibronectin antibody, human fibronectin, and goat polyclonal anti-fibronectin antibody-gold conjugate is then placed in the extraction buffer. The extraction buffer migrates up the test strip by wicking action; the polyclonal antibody-colloidal gold conjugate becomes re-suspended and migrates with the extraction buffer. If fetal fibronectin is present in the specimen, it will bind to the anti-human fibronectin colloidal gold conjugate. This complex migrates by capillary action across a membrane containing an immobilized monoclonal antibody specific to fetal fibronectin. The fetal fibronectin-anti-fibronectin-gold complex then binds to the immobilized anti-fetal fibronectin antibody, producing a visible line. If fetal fibronectin is absent from the sample, no binding occurs to the immobilized anti-fetal fibronectin antibody. Residual unbound anti-human fibronectin polyclonal antibody-gold migrates further across the membrane and binds to immobilized plasma fibronectin, providing an assay control. A positive specimen will result in two visible lines; a negative specimen will result in one visible line.

PRECAUTIONS AND WARNINGS

1. For *in vitro* diagnostic use only.
2. Read the entire directional insert before performing this test and carefully follow the instructions. Modification of the assay protocol outlined in this insert may result in erroneous results.
3. Do not mix materials from different lots.
4. Only use the applicator and extraction buffer included with the QuikCheck kit for specimen collection.

QuikCheck fFN™

5. No components from any other fFN test kits can be used with QuikCheck.
6. Do not use if applicator package integrity is compromised or if extraction buffer tube has leaked.
7. Do not use materials past their expiration dates.
8. Specimens of human origin should be considered potentially infectious. Use appropriate precautions in the collection, handling, storage, and disposal of the specimen and the used kit contents. Discard used materials in a proper biohazard container.
9. The extraction buffer contains 0.02% sodium azide, which may react with lead or copper plumbing to form potentially explosive metal azides. When disposing of this reagent, always flush the drain with large volumes of water to prevent azide build-up.
10. Handle materials with care; do not bend or compress materials.
11. The QuikCheck fFN test must be run within 15 minutes of sampling. Samples cannot be stored for later testing.
12. Care must be taken not to contaminate cervicovaginal fluid with topical agents such as lubricants, soap, disinfectants, or creams (e.g., K-Y® Jelly lubricant, Betadine® disinfectant, Monistat® cream, hexachlorophene). These substances may interfere with the specimen collection process and/or the antibody-antigen reaction of the QuikCheck fFN test.
13. The extraction buffer tube must be kept in an upright position during the assay procedure.

STORAGE AND STABILITY

All components are stable at 2° to 25°C and may be used until the expiration dates printed on the labels. Do not freeze.

REAGENTS AND MATERIALS PROVIDED

1. Sterile Applicator: One sterile polyester tipped applicator on a plastic shaft.
2. Test Strip: Membranes with immobilized mouse monoclonal anti-fetal fibronectin antibody, human fibronectin, and goat polyclonal anti-fibronectin antibody-gold conjugate.
3. Tube with Extraction Buffer: One polypropylene tube containing 1 mL extraction buffer.

MATERIALS REQUIRED BUT NOT PROVIDED

1. Test tube rack to hold extraction buffer tube
2. Timer

SPECIMEN COLLECTION AND HANDLING

Each package contains a sterile polyester tipped applicator for specimen collection. *This is the only acceptable applicator to use with this assay.* Other applicator materials interfere with the assay. Cervicovaginal secretions are obtained from the posterior fornix of the vagina. The collection process is intended to be gentle. Vigorous or forceful collection, common for microbiological cultures, is not required. During a speculum examination, prior to any examination or manipulation of the cervix or the vaginal tract, lightly rotate the applicator tip across the posterior fornix of the vagina for approximately 10 seconds to absorb cervicovaginal secretions. Subsequent attempts to saturate the applicator tip may invalidate the test. Remove the applicator and immediately perform the test as directed below. Use only the extraction buffer included with the QuikCheck kit. Samples may not be stored for later testing.

TEST PROCEDURE

1. Before collecting the patient sample, remove the tube containing the extraction buffer from the package and carefully remove the cap.
2. Collect the patient sample as instructed above with the sterile polyester tipped applicator provided. Remove the applicator and insert the tip into the tube containing the extraction buffer and mix vigorously for 10 to 15 seconds.
3. Remove as much liquid as possible from the applicator by rolling the tip against the inside of the tube. Dispose of the applicator in a manner consistent with handling potentially biohazardous materials.
4. Remove the test strip from the foil pouch making sure to handle only the labeled portion of the test strip. Insert the lower end of the test strip (dip area indicated by the arrows) into the tube containing the extraction buffer. Do not immerse the test strip further than the dip area. Do not recap tube during test strip incubation.
5. Allow the test strip to stand in the extraction buffer for 10 minutes. Immediately remove the test strip and read the result.

6. Dispose of the used test strip, extraction buffer tube, and extraction buffer in a manner consistent with handling potentially biohazardous materials.

INTERPRETATION OF RESULTS

The QuikCheck fFN test is a qualitative test. A negative result indicating the absence of fetal fibronectin will appear as one line. A positive result indicating the presence of fetal fibronectin will appear as two lines. Lines may vary in appearance from very faint to very dark. If no lines appear or if the control line does not appear, the test must be repeated.

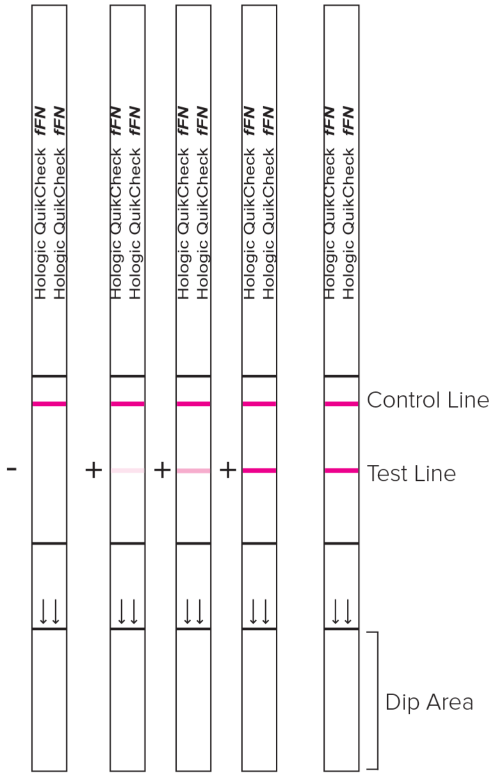

LIMITATIONS OF THE TEST

1. This assay can only be used for the qualitative detection of fetal fibronectin in cervicovaginal secretions.
2. Test results should always be used in conjunction with other clinical and laboratory data for patient management.
3. Specimens should be obtained prior to digital examination or manipulation of the cervix. Manipulations of the cervix may lead to false positive results.
4. Specimens should not be collected if the patient has had sexual intercourse within 24 hours to eliminate false positive results.
5. Patients with suspected or known placental abruption, placenta previa, or moderate or gross vaginal bleeding should not be tested.
6. Patients with cerclage should not be tested.
7. The performance characteristics of the QuikCheck fFN test are based on studies in women with singleton gestations. Performance has not been verified on patients with multiple gestations, e.g., twins.
8. The QuikCheck fFN test is not intended to be performed in the presence of rupture of amniotic membranes and rupture of amniotic membranes should be ruled out prior to conducting the test.

EXPECTED VALUES

Among symptomatic women, elevated levels (≥ 0.050 µg/mL) (1 x 10^{-7} mmol/L) of fFN between 24 weeks, 0 days and 34 weeks, 6 days indicate increased risk of delivery in ≤ 7 or ≤ 14 days from sample collection (5,11-15). Among asymptomatic women, elevated levels of fFN between 22 weeks, 0 days and 30 weeks, 6 days indicate increased risk of delivery in ≤ 34 weeks, 6 days of gestation (16-19). The cutoff of 0.050 µg/mL fFN was established in a multicenter study conducted to evaluate the association between fetal fibronectin expression during pregnancy and preterm delivery (5). The 0.050 ug/ml cutoff was standardized using purified fFN and A280 measurement ε=1.28 (21).

PERFORMANCE CHARACTERISTICS

Published Clinical Studies

- García Alonso LA, et al. Presence of fetal fibronectin (fFN) in cervico-vaginal secretion as predictor of premature labor *Ginecol Obstet Mex* 2002;70:379. The presence of fFN before 34 weeks of pregnancy predicts preterm delivery with a sensitivity, specificity, positive predictive value, and negative predictive value of 75%, 41%, 82%, and 95%, respectively, with a relative risk of 8.37 in a symptomatic population.
- Coleman, Mathew AG, et al. Fetal fibronectin detection in preterm labor: evaluation of a prototype bedside dipstick technique and cervical assessment. *Am J Obstet Gynecol* 1998;179(6):1553. Fetal fibronectin detection predicted delivery within 10 days with sensitivity, specificity, and positive and negative predictive values of 65%, 85%, 41% and 94%, respectively.
- García Alonso LA, et al. Economical impact of preterm delivery management based on fetal fibronectin results. *Ginecol Obstet Mex.* 2004 72:385-93. By determining fFN for the diagnosis of preterm labor, we obtained savings of 4,620,000 pesos in a 6 month period avoiding unnecessary treatments and hospital stay in patients with negative fFN.

Laboratory Performance

Within-Run Precision (tests run in duplicate, repeated 6 times)

Test	1	2	3	4	5	6
Number of samples	20	20	20	20	20	20
Test Positive/True Positive	12/12	12/12	12/12	12/12	12/12	12/12
Test Negative/True Negative	6/6	6/6	6/6	6/6	6/6	6/6
Test Positive/Borderline (50 ng/mL)	1/2	1/2	1/2	1/2	1/2	1/2

All 20 specimens were accurately detected with the QuikCheck fFN test.

Between-Run Precision (three test strip lots)

Tests of 12 positive samples in duplicate, 6 negative samples in duplicate and 2 borderline samples in duplicate over three lots of fFN test strips (40 tests per lot) showed 100% agreement among the lots.

Interfering Substances

Care must be taken not to contaminate the applicator or cervicovaginal secretions with lubricants, soaps, disinfectants, or creams. Lubricants or creams may physically interfere with absorption of the specimen onto the applicator. Soaps or disinfectants may interfere with the antibody-antigen reaction.

Potential interfering substances were tested at concentrations that might be reasonably found in cervicovaginal secretions. The following substances did not interfere in the assay when tested at the levels indicated.

Substance	Concentration
Ampicillin	1.47 mg/mL
Erythromycin	0.272 mg/mL
Gentamicin	0.849 mg/mL
Oxytocin	10 IU/mL
Terbutaline	3.59 mg/mL
Dexamethasone	2.50 mg/mL
$MgSO_4 \cdot 7H_2O$	1.49 mg/mL
Ritodrine	0.33 mg/mL
Prostaglandin $F_{2\alpha}$	0.033 mg/mL
Prostaglandin E_2	0.033 mg/mL
Monistat® (miconazole)	0.5 mg/mL
Indigo Carmine	0.232 mg/mL
Maternal Urine 3rd Trimester	5% (vol)
Betadine® Gel	10 mg/mL
Betadine® Cleanser	10 mg/mL
K-Y® Jelly	62.5 mg/mL
Dermicidol® 2000	25.73 mg/mL

QuikCheck fFN™ Test Kit

TROUBLESHOOTING

Q: The test line is faintly visible. Is the result positive or negative?
A: If the test line is faint but visible, the result is positive.

Q: The control line is faintly visible. Is the test valid?
A: Yes. If the control line is faint but visible, the test is valid.

Q: The test and control lines are of different intensities. Is the test valid?
A: Yes. Regardless of relative intensity, if test and control lines are visible, the test is valid.

Q: The control line is not visible. Is the test valid?
A: No. If the control line is not visible, the test is invalid. Repeat the test.

Q: The control line and/or the test line is incomplete. Is the test valid?
A: No. If the control line and/or the test line is incomplete, the test is invalid. Repeat the test.

Q: The sample was bloody. Can I use the result?
A: If the amount of vaginal bleeding is clinically judged to be minimal, the sample can be used. Patients with suspected or known placental abruption, placenta previa, or moderate or gross vaginal bleeding should not be tested. Clinical studies have demonstrated that "minimal" vaginal bleeding commonly associated with labor does not interfere with clinical interpretation of the QuikCheck fFN result.
For patients with vaginal bleeding judged to be "moderate" or "gross," the test result may be falsely positive due to the presence of small amounts of fetal fibronectin in maternal blood.

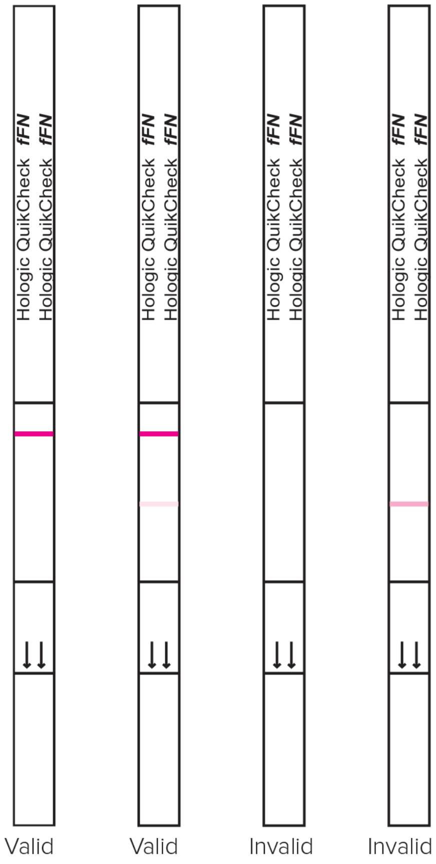

BIBLIOGRAPHY

1. American College of Obstetricians and Gynecologists. Preterm Labor. *Technical Bulletin*, Number 133, October, 1989.
2. Creasy RK, Resnick R. *Maternal and Fetal Medicine: Principles and Practice.* Philadelphia: W.B. Saunders; 1989.
3. Creasy RK, Merkatz IR. Prevention of preterm birth: clinical opinion. *Obstet Gynecol* 1990;76(Suppl 1):2S–4S.
4. Morrison JC. Preterm birth: a puzzle worth solving. *Obstet Gynecol* 1990;76(Suppl 1):5S-12S.
5. Lockwood CJ, Senyei AE, Dische MR, Casal DC, et al. Fetal fibronectin in cervical and vaginal secretions as a predictor of preterm delivery. *New Engl J Med* 1991;325:669–74.
6. Maymon R, Bahari C, Moroz C. Placental isoferritin measured by a specific monoclonal antibody as a predictive marker for preterm contraction outcome. *Obstet Gynecol* 1989;74:597–9.
7. Wasmoen TL, Coulam CB, Leiferman KM, Gleich GJ. Increases of plasma eosinophil major basic protein levels late in pregnancy predict onset of labor. *Proc Natl Acad Sci USA* 1987;84:3029–32.
8. Matsuura H, Hakomori SI. The oncofetal domain of fibronectin defined by the monoclonal antibody FDC-6: its presence in fibronectins from fetal and tumor tissues and its absence in those from normal adult tissues and plasma. *Proc Natl Acad Sci USA* 1985;82:6517-21.
9. Matsuura H, Takio K, Titani K, Greene T, et al. The oncofetal structure of human fibronectin defined by monoclonal antibody FDC-6. Unique structural requirement for the antigen specificity provided by a glycosylhexapeptide. *J Biol Chem* 1988;263:3314–22.
10. Feinberg RF, Kliman HJ, Lockwood CJ. Is oncofetal fibronectin a trophoblast glue for human implantation? *Am J Pathol* 1991;138:537-43.
11. Morrison JC, Allbert JR, McLaughlin BN, Whitworth NS, et al. Oncofetal fibronectin in patients with false labor as a predictor of preterm delivery. *Am J Obstet Gynecol* 1993;168:538–42.
12. Inglis SR, Jeremias J, Kuno K, Lescale K, et al. Detection of tumor necrosis factor-α, interleukin-6, and fetal fibronectin in the lower genital tract during pregnancy: Relation to outcome. *Am J Obstet Gynecol* 1994;171:5–10.

13. Iams J, Casal DC, Goodwin TM, Kreaden US, et al. Fetal fibronectin improves the accuracy of diagnosis of preterm delivery. *Am J Obstet Gynecol* 1995;173:141–5.

14. Burrus DR, Ernest JM, Veille JC. Fetal fibronectin, interluekin-6, and C-reactive protein are useful in establishing prognostic sub-categories of idiopathic preterm labor. *Am J Obstet Gynecol* 1995;173:1258–62.

15. Bartnicki J, Casal DC, Kreaden US, Saling E, Vetter K. Fetal fibronectin in vaginal specimens predicts preterm delivery and very low birth weight infants. *Am J Obstet Gynecol* 1996;174:971–4.

16. Lockwood CJ, Wein R, Lapinski R, Casal D, et al. The presence of cervical and vaginal fetal fibronectin predicts preterm delivery in an inner-city obstetric population. *Am J Obstet Gynecol* 1993;169:798–804.

17. Leeson SC, Maresh MJA, Martindale EA, Mahmood T, et al. Detection of fetal fibronectin as a predictor of preterm delivery in high risk asymptomatic pregnancies. *Br J Obstet Gynecol* 1996;103:48–53.

18. Goldenberg RL, Mercer BM, Meis PJ, Copper RL, et al. The preterm prediction study: fetal fibronectin testing and spontaneous preterm birth. *Obstet Gynecol* 1996;87:643–8.

19. Morrison JC, Naef RW, Botti JJ, Katz M, et al. Prediction of spontaneous preterm birth by fetal fibronectin and uterine activity. *Obstet Gynecol* 1996;87:649–55.

20. McGregor JA. Salivary estriol as risk assessment for preterm labor: a prospective trial. *Am J Obstet Gynecol* 1995;173:1337–42.

21. Yamada KM. “Fibronectin and Other Structural Proteins.” in *Cell Biology of Extracellular Matrix,* Ed ED Hay. 1st ed. New York: Plenum Press, 1981;95-114.

TECHNICAL SERVICE AND ORDERING INFORMATION

Contact your local Hologic representative or call:

Tel: 00800 800 29892

For additional contact information, go to www.ffntest.com

©2017 Hologic, Inc. All rights reserved.

Hologic, QuikCheck and/or associated logos are trademarks and/or registered trademarks of Hologic, Inc. and/or its subsidiaries in the United States and/or other countries. All other trademarks, registered trademarks, and product names are the property of their respective owners.

⚠	Caution, consult accompanying documents	EC REP	Authorized Representative in the European community	⌛	Use by
REF	Catalogue number	IVD	*In Vitro* diagnostic medical device	2°C / 25°C	Temperature limitation: 2°–25°C
LOT	Batch code		Manufacturer		Do not reuse

Hologic, Inc. • 1240 Elko Drive • Sunnyvale, CA • 94089-2212 • USA
1 (888) PRETERM • +1 (508) 263-2900 • www.hologic.com

EC REP Hologic Ltd. • Heron House, Oaks Business Park, Crewe Road, Wythenshawe, Manchester, M23 9HZ UK • +44 (0)161 946 2206

QuikCheck fFN™
