# Supplementary material for: Benefits of Premaquick® Combined Detection of IL-6/Total IGFBP-1/Native IGFBP-1 to Predict Preterm Delivery
Source: J Clin Med. 2023 Sep 1;12(17):5707. doi: 10.3390/jcm12175707 (PMC10488604; doi:10.3390/jcm12175707)
Supplement: Supplementary file 1 [file jcm-12-05707-s001.zip › Supplementary File S2.pdf]

PREMAQUICK®

Ref : 1090009

EN

RAPID TEST FOR THE PREDICTION OF IMMINENT DELIVERY

For professional in vitro diagnostic use.

CE

1 I INTENDED USE

PREMAQUICK® is a rapid multiparameter test for the in vitro detection of IGFBP-1 (Insulin-like Growth Factor-Binding Protein 1), fragmented forms of IGFBP-1 and IL-6 (Interleukin 6) in vaginal secretions. PREMAQUICK® is intended for assessing the risk of imminent birth between 22 and 37 (+6 days) weeks of amenorrhea in women who present with threatened preterm labour (PTL). IGFBP-1 is a marker of cervical ripening. Its presence in vaginal secretions in the absence of ruptured membranes shows a significant lysis of the decidual cells of the cervix and a diffusion of amniotic fluid during contractions. The presence of fragmented forms of IGFBP-1 indicates a significant local proteolytic activity and fetal stress caused by contractions. The third marker, IL-6, is a marker of inflammation or infection of the amniotic cavity and the cervicovaginal area. By combining the biomarkers of myometrium activity, ripening of the cervix and inflammation/infection, PREMAQUICK® incorporates the principal pathogenic mechanisms for preterm labour, which allows us to predict or rule out the risk of labour within 7 to 14 days. This test is intended for in vitro diagnosis use exclusively by health professionals.

2 I INTRODUCTION

Each year, 15 million babies are born prematurely in the world. This leads to 1 million neonatal deaths (WHO, 2012). In 50% of cases, premature births are preceded by threatened preterm labour (PTL), pathology that is associated with cervical changes and regular, painful uterine contractions. However, only 20% of patients with threatened PTL give birth prematurely. It is therefore essential to accurately predict imminent birth in the presence of threatened PTL in order to consider therapeutic intervention (corticosteroids, tocolytics), hospitalisation, or for patients who really need it, being transferred to a specialised maternity hospital.

Measuring transvaginal cervical length via the endovaginal route is the most commonly used method but its performance to predict labour within 7 days are limited (sensitivity 78.3% and specificity 71% at the threshold of 25 mm). What is more, foetal fibronectin, the most documented biomarker for preterm labour, shows a good negative predictive value but its positive predictive value remains limited (in the range of 15% to 48% to predict labour within 7-10 days).

3 I TEST PRINCIPLE

PREMAQUICK® is a rapid lateral flow test composed of three strips allowing the detection of native IGFBP-1 (IGFBP-1), total IGFBP-1 (native + fragmented, IGFBP-1 T) and IL-6. For the detection of native IGFBP-1, the test uses a pair of monoclonal antibodies directed against IGFBP-1. The first antibody, the capture antibody, is immobilised on the nitrocellulose membrane at the level of the test line (T). During the test, if the antigen is present in the patient sample it reacts with the monoclonal antibodies conjugated with gold particles, present in a non-visible part of the test. The complex thus formed migrates and interacts with the antibodies fixed in the test lines. Then a purple line appears. A similar principle is used to detect IL-6 with a pair of monoclonal anti-IL-6 antibodies. To detect total IGFBP-1, three monoclonal antibodies are used: a monoclonal antibody for capture and two conjugated antibodies, one directed against IGFBP-1 and the other directed against a specific portion of the N-terminal of the same protein.

4 I MATERIALS

Materials provided

- Test cassettes packed in individual pouch containing a desiccant.
- Vaginal swabs flocked with Sterile Nylon CE 0123.
- Dropper bottles with buffer. DIL
- Instructions for use.
- Patient cards.

Materials required but not provided

- Stop watch with alarm.

5 I STORAGE AND STABILITY

PREMAQUICK® tests are wrapped in aluminium packaging with a desiccant. The tests should be stored in a dry place at a temperature between 2°C and 30°C.

This test is stable until the expiry date printed on the aluminium packaging. The cassette should be kept away from damp. The test must remain in its sealed pouch until use.

6 I PRECAUTIONS

- For *in vitro* diagnostic use only.
- For best results, carefully follow the procedure and the storage instructions.
- Do not open the aluminium packaging until it is at room temperature to prevent condensation. Damp and high temperatures may affect results.
- Wear protective clothing, disposable gloves and protective glasses when handling potentially infectious materials and when performing the test.
- Patient samples and all other materials should be considered and treated as potentially infectious. Dispose of the different elements of the test and the samples according to the applicable procedures for potentially infectious waste.
- Avoid splatters and formation of aerosols. Clean all spilled liquid with an appropriate disinfectant.
- The tests, swabs and tubes are intended for single use. Do not reuse the cassettes, swabs or dilution tubes.
- Do not combine or mix up the reagents of kits or different batch numbers.
- Do not use a test if the aluminium packaging is opened or damaged.
- This test is only intended for visual reading and interpretation.

7 I SPECIMEN COLLECTION AND HANDLING

The sample should be taken before any vaginal examination or endovaginal ultrasound with the sterile nylon swab that comes with the test.

The procedure is as follows:

- Remove the swab from its packaging.
- Use a speculum to introduce the swab into the vagina and collect the sample at the level of the superior fornix by holding it against the vaginal wall for 15 seconds.
- Place the swab into the extraction tube immediately after taking the sample (see step #3 in the test procedure described below).

The tube can be kept for a maximum of 6 hours at room temperature or refrigerated at 4°C before performing the test.

8 I TEST PROCEDURE

1. Allow the complete kit and specimens to stabilize at room temperature (15°C-30°C) before performing the assay. Open the foil pouch, remove the test cassette from its packaging and place it on a flat, horizontal surface.
2. Unscrew the black cap to open the dropper bottle and place it on a horizontal level surface.
3. Immerse the swab into the bottle and rotate for a dozen or so seconds. Press the sides of the tube to extract the most liquid possible from the swab. Then remove the swab or break its end off in the bottle.
4. Close the bottle and shake. Open the upper part of the cap (transparent plug) to access the drops. Hold the bottle vertically and add 3 drops of the diluted sample into the 3 sample wells of the cassette by applying slight pressure to the sides of the tube. Avoid adding air bubbles to the cassette sample well and tipping the liquid into the result reading window.
5. Start the timer. As the test progresses a reddish colour migration front appears and migrates along the membrane.
6. Read the result after 10 minutes of migration. Strongly positive results may be visible earlier. Do not interpret any test band appearing 15 minutes after the sample is deposited in the cassette.
7. Dispose of the components of the test and the swab according to the procedure applicable to potentially infectious waste.

9 I RESULTS

The test is read visually. The results are interpreted as negative, positive or invalid depending on the appearance of coloured lines in the procedure control area (C) and test areas (T) for the 3 parameters. The presence of three control bands (C) in the result reading windows is necessary for the test to be valid.

The interpretation of the presence or absence of the test band T (even of weak intensity) for each parameter is written as a score.

- A score of 3 is attributed in the presence of a test band T for the IGFBP-1 N parameter,
- A score of 2 is attributed in the presence of a test band T for the IGFBP-1 parameter,
- A score of 1 is attributed in the presence of a test band T for the IL-6 parameter,
- The absence of a test band T in all 3 result reading windows corresponds to a score of 0 for each of the 3 parameters.

The scores obtained for each parameter are then summed to determine the total score:

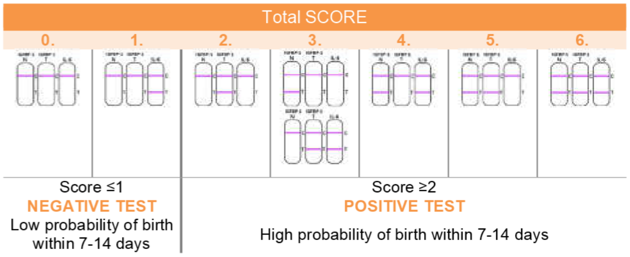

INVALID TEST:

The absence of at least one control band (C) in the result reading window even in the presence of a (T) band invalidates the results of the test. Results from a test without the control line should not be taken into consideration. Review the procedure and repeat it with a new device. If the problem persists, contact your local distributor.

10 I QUALITY CONTROL

Internal controls:

A control line is used as an internal control of the test procedure, its appearance indicates that the sample volume used is sufficient and that the procedure was followed correctly.

External controls:

External controls are not supplied with this kit. Good laboratory practice recommends the use of external controls. It is recommended to check each new batch or shipment. Each laboratory should establish its own control planning.

11 I LIMITATIONS OF PROCEDURE

1. As for any diagnostic test, the test result must be correlated with clinical results.
2. In the presence of a significant amount of blood in the vaginal secretion sample leading to a significant red coloring of the bud of the swab, a positive result should be interpreted with caution.
3. Sperm or semen can create false results and should therefore be avoided.

BIOSYNEX SA
22 Boulevard Sébastien Brant
67400 ILLKIRCH-GRAFFENSTADEN
Tel FR : +33 3 88 78 78 87
Fax : +33 3 88 78 76 78
Tel clients Export : +33 3 88 77 57 52
client.pro@biosynex.com
www.biosynex.com
export@biosynex.com

12 I PERFORMANCE

Analytical sensitivity:

- The detection limit of the test strip for native IGFBP-1 is 5 ng/mL of recombinant native IGFBP-1.
- The detection limit of the test strip for total IGFBP-1 is 10 ng/mL of recombinant native IGFBP-1 and 10 ng/mL of an N-terminal fragment of IGFBP-1.
- The detection limit of the test strip for IL-6 is 0.25 ng/mL of recombinant IL-6.

Clinical study:

A prospective multicentre test was conducted on 97 women who presented with threatened PTL. The PREMAQUICK® test was performed from vaginal secretions taken using a speculum before a vaginal exam as performed.

The patients of the study were followed for 14 days after their admission and the performance of the PREMAQUICK® test for predicting labour within 2/7/24 days of the test were calculated.

The following results were obtained:

	≤ 2 days	≤ 7 days	≤ 14 days
Sensitivity	94.7% (74.0/-99.9)	93.5% (78.6/-99.2)	94.3% (80.8/-99.3)
Specificity	78.2% (67.4/-86.8)	90.9% (81.3/-96.6)	96.8% (88.8/-99.6)
Positive predictive value (PPV):	51.4% (34.0/-68.6)	82.9% (66.4/-93.4)	94.3% (80.8/-99.3)
Negative predictive value (NPV):	98.4% (91.3/-99.9)	96.8% (88.8/-99.6)	96.8% (88.8/-99.6)

What is more, the probability of labour within 2/7/14 days in the case of a triple positive (score=6) is 66.7%/95.8%/95.8% while the probability of no labour within 2/7/14 days in the case of a triple negative (score=0) is 100%/98%/98%.

13 I LITERATURE

1. Rutanen EM, Pekonen F, Kärkkäinen T. Measurement of insulin-like growth factor binding protein-1 in cervical/vaginal secretions: comparison with the ROM-check Membrane Immunoassay in the diagnosis of ruptured fetal membranes. Clin Chim Acta. 1993 Jan 31;214(1):73-81.
2. Lange M, Chen FK, Wessel J, Buscher U, Dudenhausen JW. Elevation of interleukin-6 levels in cervical secretions as a predictor of preterm delivery. Acta Obstet Gynecol Scand. 2003 Apr;82(4):326-9.
3. Hills FA, Iles RK, Sullivan MH. Differential proteolysis of insulin-like growth factor binding protein-1 (IGFBP-1) in pregnancy. J Perinat Med. 2013 May;41(3):241-9. doi: 10.1515/jpm-2012-0086.
4. Lee J, Lee SM, Oh KJ, Park CW, Jun JK, Yoon BH. Fragmented forms of insulin-like growth factor binding protein-1 in amniotic fluid of patients with preterm labor and intact membranes. Reprod Sci. 2011 Sep;18(9):842-9. doi: 10.1177/1933719111399927. Epub 2011 Mar 18.
5. Woodworth A, Moore J, G'Sell C, Verdoes A, Snyder JA, Morris L, Wares C, Grenache DG, Gronowski AM. Diagnostic accuracy of cervicovaginal interleukin-6 and interleukin-6:albumin ratio as markers of preterm delivery. Clin Chem. 2007 Aug;53(8):1534-40.
6. March of Dimes, PMNCH, Save the children, WHO. Born Too Soon: The Global action report on preterm Birth. Eds CP Howson, MV Kinney, JE Lawn. World health organization. Geneva, 2012
7. Sotiriadis A, Papatheodorou S, Kavvadias A, Makrydimas G. Transvaginal cervical length measurement for prediction of preterm birth in women with threatened preterm labor: a meta-analysis. Ultrasound Obstet Gynecol. 2010 Jan;35(1):54-64. doi: 10.1002/uog.7457.
8. Compan C, Rossi A, Piquier-Perret G, Delabaere A, Vendittelli F, Lemery D, Gallot D. Prédiction de la prématurité en cas de menace d'accouchement prématuré : revue de la littérature. Journal de Gynécologie Obstétrique et Biologie de la Reproduction 2015 ;44 :740-751

SYMBOLS

	Attention, see instruction for use		Tests per kit	REF	Catalog number
IVD	For *in vitro* diagnostic use only	2°C 30°C	Store between 2-30°C		Do not reuse
	Do not use if package is damaged	LOT	Lot number		Expiry
	Manufacturer	DIL	Buffer		

IFU_1090009_EN_V03202010R01
Date of last revision : 10/2020

BIOSYNEX SA
22 Boulevard Sébastien Brant
67400 ILLKIRCH-GRAFFENSTADEN
Tel FR : +33 3 88 78 78 87
Fax : +33 3 88 78 76 78
Tel clients Export : +33 3 88 77 57 52
client.pro@biosynex.com
BIOSYNEX EASY DIAGNOSTICS FOR LIFE
www.biosynex.com
export@biosynex.com
